# The Locus Preservation Hypothesis: Shared Linguistic Profiles across Developmental Disorders and the Resilient Part of the Human Language Faculty

**DOI:** 10.3389/fpsyg.2017.01765

**Published:** 2017-10-13

**Authors:** Evelina Leivada, Maria Kambanaros, Kleanthes K. Grohmann

**Affiliations:** ^1^Language and Culture, UiT-The Arctic University of Norway, Tromsø, Norway; ^2^Rehabilitation Sciences, Cyprus University of Technology, Limassol, Cyprus; ^3^Cyprus University of Technology, Limassol, Cyprus; ^4^English Studies, University of Cyprus, Nicosia, Cyprus

**Keywords:** distributed morphology, grammatical marker, linguistic phenotype, syntax, Autism spectrum disorders (ASD), Down Syndrome, specific language impairment (SLI)

## Abstract

Grammatical markers are not uniformly impaired across speakers of different languages, even when speakers share a diagnosis and the marker in question is grammaticalized in a similar way in these languages. The aim of this work is to demarcate, from a cross-linguistic perspective, the linguistic phenotype of three genetically heterogeneous developmental disorders: specific language impairment, Down syndrome, and autism spectrum disorder. After a systematic review of linguistic profiles targeting mainly English-, Greek-, Catalan-, and Spanish-speaking populations with developmental disorders (*n* = 880), shared loci of impairment are identified and certain domains of grammar are shown to be more vulnerable than others. The distribution of impaired loci is captured by the *Locus Preservation Hypothesis* which suggests that specific parts of the language faculty are immune to impairment across developmental disorders. Through the Locus Preservation Hypothesis, a classical chicken and egg question can be addressed: Do poor conceptual resources and memory limitations result in an atypical grammar or does a grammatical breakdown lead to conceptual and memory limitations? Overall, certain morphological markers reveal themselves as highly susceptible to impairment, while syntactic operations are preserved, granting support to the first scenario. The origin of resilient syntax is explained from a phylogenetic perspective in connection to the “syntax-before-phonology” hypothesis.

## Introduction

In his seminal book *The Biological Foundations of Language*, Eric Lenneberg made the following observation when comparing different states of verbal behavior:

Some aphasic symptoms *bear certain similarities* to the common derangements of speech and language seen in individuals in good health under conditions of mental exhaustion or states of drowsiness […]. Clinically, *we may encounter an almost kaleidoscopic combination of idiosyncratic failure or sparing of particular skills* which renders precise correlations between pathological anatomy and pathological verbal behavior very difficult (Lenneberg, 1967, p. 222; emphasis added).

Almost 40 years later, Phillips (2005) observed, when comparing the underpinnings of various developmental language impairments, that some aspects of language such as morphosyntactic difficulties associated with tense inflection appear to be affected across pathologies with different genetic causes (e.g., Specific Language Impairment, autism, Williams Syndrome, Down Syndrome, fragile X syndrome). Similarly, many studies have identified overlaps at the phenotypic level among different disorders: Leyfer et al. (2008) and Durrleman and Delage (2016) for autism spectrum disorder (ASD) and specific language impairment (SLI), Perovic et al. (2013) for ASD and Williams Syndrome, Dykens et al. (2011) for Prader-Willi syndrome and ASD, Eadie et al. (2002) for Down Syndrome (DS) and SLI, and Bishop (2014b) for anterior aphasia and SLI.

Overlaps in the behavioral profile of populations with different diagnoses have led to the claim that variation across phenotypes (i.e., breakdowns) is constrained in a way that renders some aspects of language processing—or more generally, cognition—more vulnerable in all pathological conditions, while others are consistently spared across individuals and conditions, both acquired and developmental (Phillips, 2005; Glisky, 2007; Kambanaros and van Steenbrugge, 2013; Benítez-Burraco and Boeckx, 2014; Leivada, 2014, 2015; Kambanaros and Grohmann, 2015; Tsimpli et al., 2017a). This high vulnerability of certain aspects of language is possibly the result of brain network organization. Studies on the distribution of lesions at the human connectome suggest that hubs are more likely to be anatomically abnormal than non-hubs across many, or possibly all, brain disorders because of their high centrality (van den Heuvel and Sporns, 2013; Crossley et al., 2014). Furthermore, multiple theoretical perspectives and neuroimaging research are addressing outstand ing questions about the nature and extent of brain connectivity aberrations in SLI vs. autism (Verhoeven et al., 2012) and DS (Anderson et al., 2013).

At the same time, studies disagree about the status of a grammatical marker as vulnerable or not, even when reporting on the competence and/or performance of speakers of the *same* language; for example, see Manika et al. (2010) for the greater variability that exists between studies that report on the status of clitics in Greek SLI. This phenotypic variability across linguistic profiles is observed even *within* one pedigree, where affected members share a diagnosis, as Bartha-Doering et al. (2016) have shown for SLI. One could, of course, argue that this is due to the character of SLI as a disorder that relies on an exclusionary diagnosis. In other words, because the criteria for diagnosing SLI are exclusionary (Reilly et al., 2014), this inevitably forms a largely heterogeneous disorder with diverse subtypes that encompass very different populations. However, the same phenotypic variability can be observed in impaired phenotypes that rely on an inclusionary diagnosis. For instance, Fowler (1995) notes that there is tremendous variability with respect to language function in individuals with DS. Lecavalier (2014) raises the same observation for ASD. Overall, this variability could be the result of variable expressivity. Individuals that carry a pathogenic variant of a gene can be impaired in a non-uniform fashion and this may result in different cognitive subtypes within an impaired phenotype (see Geschwind, 2011 for a review of variable expressivity in ASD). In this context, it becomes clear that the attained performance is not necessarily homogeneous even among people that share the same developmental disorder and speak the same language.

The picture painted by this brief overview involves a paradox. Although specific markers are highly vulnerable and as such prone to impairment across disorders, there still exists a lot of variability in terms of the attested impairment both across and within disorders. Phillips (2005) calls this state of affairs “a clear puzzle” and presents it in the following way:

On the one hand, the effects of specific genetic disorders on language appear to be surprisingly *nonspecific*. Similar aspects of language appear to be impacted across a variety of disorders with different genetic causes. On the other hand, the effects of genetic disorders on language are *highly specific*. [Developmental language impairments] appear to *selectively target* certain subparts of language while sparing others (Phillips, 2005, p. 79; emphases added).

This picture might even include derangements of speech in healthy, neurotypical adults, as noted in Lenneberg (1967) and quoted above.

In the present work, it is argued that the solution to Phillips' puzzle requires (i) a fine-grained analysis of loci of variation across different developmental impairments, which is (ii) situated within linguistic frameworks that put forth a clear division of labor between the different parts of grammar, and (iii) approached from a cross-linguistic perspective. In what follows we present work on (i), on (ii), and in parts on (iii), through comparing the linguistic profiles of three different types of developmental disorders (SLI, ASD, and DS) in speakers of two varieties of Greek (Stand ard Modern Greek and Cypriot Greek), English, Spanish, and Catalan.

**Figure 1:**
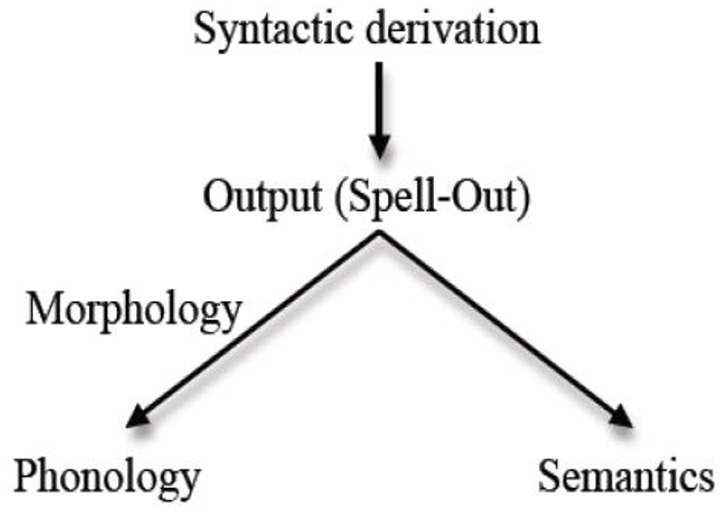
Architecture of a minimalist/distributed morphology grammar (Bobaljik, 2017, p. 1).

We employ the layout of grammar put forth in the framework of Distributed Morphology (Halle and Marantz, 1993; Harley and Noyer, 1999; Figure 1) in order to identify which aspects of language feature the various loci of impairment. This model does not enhance the testability of our argument—but it does facilitate organizing the distribution of impaired markers across levels of linguistic analysis in a transparent way. In this framework (and minimalism at large), “syntactic derivation” refers to operations in syntax proper, the outcome of which feeds the other levels of analysis: phonology (via Phonetic/Phonological Form) and semantics (via Logical Form). Spell-out is an instruction to transfer this outcome to the next stage of operations.

By using a theoretical linguistics model as a vehicle for cartographing vulnerable loci across disorders, we establish an interdisciplinary connection between theoretical linguistics and the clinical aspects of cognitive neuroscience. Such interdisciplinary bridges are crucial in the study of language perhaps today more than ever, for it has been recently argued that linguistics, once seen the key player in the field of cognitive science, has seen its influence on closely allied disciplines fade away over the last years (Ferreira, 2005; Hagoort, 2014). However, one should not ignore the considerable body of literature that establishes interdisciplinary bridges in a way that shows how notions and primitives from theoretical linguistics can contribute to the study of neuroscience and other closely allied disciplines (Marantz, 2005; Sprouse and Almeida, 2013; Leivada, 2015). Against this background, the second aim of the present work is to offer a concrete example of how models of grammar in theoretical linguistics can inform the study of the brain through the investigation of pathological phenotypes. The study of the latter offers a unique perspective into the “physical mechanisms of the brain that correspond to the various domains of grammar and its structure” (Terzi, 2005, p. 111).

## Methods

The case reports presented in the following are the result of extensive database searching through PubMed, SCOPUS, ScienceDirect, and Google Scholar, as well as probing individual journals for results retrieved by searches for any combination of the terms “primary/specific language impairment,” “autism spectrum disorder(s),” “Down('s) syndrome,” “linguistic phenotype,” “impaired/atypical phonology/morphology/syntax/semantics/pragmatics,” “word retrieval in SLI/ASD/DS,” and “linguistic impairment/disorder.” Our searches were constrained in terms of a time frame that covered the last two decades and in terms of language groups (Greek, English, Catalan, and Spanish). In choosing these language combinations, our aim was to cover both monolingual (Stand ard Modern Greek, English, Spanish) and bilectal/bilingual populations (Stand ard Modern Greek–Cypriot Greek, Spanish–Catalan) and languages with rich morphology. A cross-linguistic perspective is likely to shed light to the vulnerable parts of language in a way that goes beyond language-specific particularities. If any, it is the cross-linguistic study of the pathologies under investigation that has the potential to uncover the common denominator and the factors that distinguish children with a pathological linguistic profile from their typically developing peers (Leonard, 2014).

### Specific language impairment (SLI)

Specific language impairment is a developmental disorder marked by limitations in the process of language development. It is usually assumed that these limitations occur in the absence of neurological damage such as hearing impairment, motor skills disorder, and low non-verbal IQ, and in the presence of otherwise typical cognitive development (Leonard, 1998). SLI is largely heterogeneous and many distinct subtypes have been identified in the literature. Two common SLI subtypes are typical SLI and pragmatic language impairment (Bishop, 2004): the former refers to those cases that involve problems with grammatical development (e.g., omission of paste tense morphemes in English), sometimes referred to as G(rammatical)-SLI (van der Lely, 2005) or Sy(ntactic)SLI (Friedmann and Novogrodsky, 2008), while the latter indicates social communication problems (e.g., lack of coherence in conversation). In some studies, these linguistic limitations have been grounded in cognition rather than language *per se* (e.g., working memory limitations; Gathercole and Baddeley, 1990; Dodwell and Bavin, 2008), leading to the conclusion that SLI is not really specific to language, as its name suggests (Engel de Abreu et al., 2014). This has led to serious debates, and no consensus, in the literature on terminology for defining SLI (Bishop, 2014a; Reilly et al., 2014). Table 1 presents 44 studies that feature different language groups and sets of tasks. These studies have been selected so as to include representation of all domains of impairment that have been proposed in the relevant literature on SLI.

**Table 1:** SLI studies across different language groups and sets of tasks.

**Study**	**Task(s)**	**Results**	**Domain(s) of impairment**
**(A) OVERVIEW OF STUDIES ON SLI: ENGLISH**			
O'Hara and Johnston, 1997	Acting out auditory stimuli with toys	Lower accuracy than controls in acting out the meaning of the stimuli	Processing limitations
Loeb et al., 1998	Verb elicitation and causative alternation	Problems with (i) the production of passive forms and (ii) the transitive–intransitive alternation	Syntax
Montgomery and Leonard, 1998	On-line word recognition and off-line grammaticality judgment	Sensitivity to the presence of high-substance inflection	Morphophonology due to general processing capacity limitation (Surface Hypothesis)
Leonard et al., 2003	Elicited production of past progressive *was/were* ±*ing*, present progressive *is/are* ±*ing*, third person singular −*s* in habitual action contexts, modal *can* in ability/possibility contexts	Affected tense marking	Morphology
Montgomery, 2004	Picture-pointing sentence comprehension and non-word repetition	Poor long non-word repetition and poor comprehension of normal-rate sentences	Phonological working memory
Bishop and Donlan, 2005	Story retelling	Poorer story recall than controls	Difficulties in encoding and remembering meaningful sequences of events
Schuele and Dykes, 2005	Conversational samples	Omissions of (i) infinitival *to*, (ii) relative markers and pronouns, (iii) *wh*-pronouns in embedded *wh*-clauses	Delayed emergence of complex syntax
Lin, 2007	Corpus study	Errors in tense marking and agreement Intact case marking and A-movement	Agreement marking errors as spell-out errors
Marinis and van der Lely, 2007	Cross-modal picture priming	90% accuracy in question comprehension	Syntactic filler–gap dependencies
Marshall et al., 2009	Chunking (receptive task, expressive task), focus (receptive task, expressive task), long-item (discrimination-receptive task, imitation-expressive task)	Failure to disambiguate when prosody is required to interact with syntax/discourse Ability to imitate or discriminate prosodic structures without reference to meaning	Integration of prosody in overall processing
Riches et al., 2010	Sentence repetition	Errors on the more complex object relative clauses	Syntax due to short-term memory limitations
Owen van Horne and Lin, 2011	Conversational and narrative samples	Use of cognitive state verbs, especially high-frequency ones, but reduced combination of low-frequency verbs with complement clauses compared to controls	Lexical knowledge limitations
Claessen and Leitão, 2012	Auditory lexical discrimination, phoneme deletion	Lower quality phonological representations	Phonology
Riches, 2012	Sentence repetition	Errors in sentence repetition and difficulties in delayed repetition	Syntactic representation and maintenance of long-term memory representations in short-term memory
Leonard et al., 2013	Sentence comprehension	Intact syntactic and lexical representations Reduced accuracy in high-demand processing	Processing limitations
Marinis and Saddy, 2013	Off-line picture selection, self-paced listening and picture verification	Difficulties in reanalyzing thematic roles in both actives and passives	Processing limitations
Tomas et al., 2015	Elicited production of past tense –*ed*, third person singular –*s*, possessive marker –*s*	Difficulties with syllabic allomorphs	Morphophonology
**(B) OVERVIEW OF STUDIES ON SLI: STANDARD MODERN GREEK**			
Clahsen and Dalalakis, 1999	Spontaneous speech	Subject–verb agreement at chance level due to overproduction of 3rd person Intact past tense markings	Morphology
Tsimpli and Stavrakaki, 1999	Spontaneous speech	Omission of direct object and definite articles Mastery of strong pronouns and indefinite articles	Grammar
Kateri et al., 2005	Picture naming	Non-target production of certain phonemes	Phonology
Manika et al., 2010	Elicited production	At ceiling production of direct object clitics and definite articles	—
Mastropavlou, 2010	Sentence completion	Tense marking errors which are less featured on morphophonologically salient forms	Morphology
Stavrakaki and van der Lely, 2010	Elicited production, picture selection	Poorer production and comprehension of object clitics compared to controls	Syntax
Mastropavlou and Tsimpli, 2011	Spontaneous speech	Complementizer omission (for some types of complementizers)	Morphophonology (omissions as spell-out errors)
Lalioti et al., 2016	Non-word repetition, elicited production, off-line grammaticality judgment, on-line self-paced listening	Intact subject–verb agreement, slower reaction times compared to controls, lower performance in the grammaticality judgment task, limited verbal short-term memory	Verbal short-term memory
Tsimpli et al., 2017b	Clitic production, picture-based story retelling	Lower accuracy in first person clitics compared to controls	Theory of Mind deficits
**(C) OVERVIEW OF STUDIES ON SLI: STANDARD MODERN GREEK AND CYPRIOT GREEK**			
Petinou and Terzi, 2002	Spontaneous speech	Clitic displacement errors Intact clitic production	Grammar
Spanoudis et al., 2007	Elicited production	Poor knowledge of mental state verbs, poor inference drawing	Semantics Pragmatics
Kambanaros, 2013, 2014; Kambanaros et al., 2014	Picture naming, connected speech, naming of semantically and phonologically complex verbs	Naming errors Better retrieval of nouns than verbs No difference between noun and verb use in connected speech Greater difficulty naming semantically complex but not phonologically complex verbs	Access to phonological representations
Theodorou and Grohmann, 2015	Elicited production of clitics	Intact clitic production	—
**(D) OVERVIEW OF STUDIES ON SLI: SPANISH**			
Bedore and Leonard, 2001	Elicited production of present first/third person singular/plural, past first/third person singular/plural, infinitive, definite articles, indefinite articles, direct object clitic pronouns, noun plural inflections, and adjective agreement inflections	Limited use of inflectional morphemes (e.g., agreement markers and direct object clitics)	Morphology
Restrepo and Gutierrez-Clellen, 2001	Interview, picture description, story retelling	Errors in unstressed definite articles	Morphophonology (Surface Hypothesis)
Katsos et al., 2011	Acceptability judgment task	Difficulty with employing the maxim of quantity	Pragmatics
Grinstead et al., 2013	Spontaneous speech, repetition task	Affected tense markings	Morphology
Andreu et al., 2016	Sentence comprehension	Intact accessing of the semantic information of verbs	—
Gavarró and Cantú-Sánchez, 2016	Vocabulary retrieval, non-word repetition, elicitation of morphology and syntax	Intact vocabulary, non-word repetition, determiners, sentence repetition, subject and object relatives, preposition, nominal agreement, and pluralization	—
**(E) OVERVIEW OF STUDIES ON SLI: SPANISH AND CATALAN**			
Aguilar-Mediavilla and Serra-Raventós, 2006	Interview	Delay in the acquisition of segments, complex syllabic structures, and differences compared to controls in the production of various segments	Phonology
Sanz-Torrent et al., 2008	Spontaneous speech	Verb inflection	Morphology (Surface Hypothesis)
Andreu et al., 2013	Picture description	Omissions of obligatory arguments (themes) as complexity increased	Limited semantic representations
			Processing limitations
Aguilar-Mediavilla et al., 2014	Navarra Oral Language Test–Revised (PLON-R: Prueba del Lenguaje Oral de Navarra–Revisada)	Reduced phonological awareness and memory, letter identification, and semantic comprehension	Phonology
			Semantics
			Processing limitations
Buil-Legaz et al., 2016	Referential communication task	Less informative messages compared to controls	Pragmatics
			Processing limitations
Gavarró and Lite, 2015 [for Catalan]	Truth value judgment task	Intact semantics of quantifiers	—

Table 1 identifies all domains of grammar as potentially impaired in SLI populations. However, a closer look at the relevant results suggests that only *some* domains of grammar are truly atypical. It is clear that many studies report problems in morphophonology or pragmatics as well as general processing limitations. The nature of the impairment is less clear, though, in studies that argue in favor of a problem in the syntactic domain. Before showing why, we understand syntax as (the iterative application of) the operations (internal and external) Merge and Agree, following the definitions of Chomsky (2001). Many of the studies reviewed refer to omissions of agreement markers or failure to establish agreement/binding relations between different components of structure when talking about impaired syntax (e.g., Clahsen and Dalalakis, 1999; Tsimpli and Stavrakaki, 1999; Lin, 2007), and we follow the assumption that syntax indeed hosts these relations. The reason is that it is necessary to revisit the *results* of these studies—and explain in what sense they are not truly making a case for a deficient syntax—, instead of evoking an argument that dismisses the syntactic nature of these relations (binding/agreement) on theoretical grounds (e.g., by suggesting that Agree takes place post-syntactically, so when a study reports agreement errors, this does not concern syntax in the first place).

Returning to the studies in Table 1, Loeb et al. (1998) claim that the performance of the SLI group demonstrates a problem in syntax—yet their difference from controls is evident only in passives and *some* types of transitive–intransitive alternation responses but not in all. If the syntactic mechanisms responsible for this production were broken, how is it possible that they function for some types of stimuli? This variation suggests that these mechanisms are present and operative, but the overt realization of their output (“externalization”) might be affected depending on many factors such as the complexity of the task demand s (e.g., working memory overload).

Passivization is a classic example of the so-called syntactic deficit. As Penke (2015) notes, most language-impaired individuals would understand better a canonical SVO structure (e.g., “John kissed Mary”) compared to object clefts (e.g., “It is Mary who John kissed”) or passives (e.g., “Mary was kissed by John”). She notes that language-impaired individuals often misinterpret such structures by interpreting the first NP encountered as AGENT (as in the canonical SVO) instead of THEME. However, this is not a very concrete indication of a syntactic deficit for the following reason: The same mistake (i.e., the strategy to interpret the first NP of a clause as AGENT) is *regularly observed* in control groups that do not have any language impairment whatsoever (Penke, 2015). In other words, the same strategy is employed by healthy neurotypical subjects that have an intact, fully functional syntactic domain. This probably happens because the human parser establishes a threshold for the interpretation of each chunk of input. As noted in Leivada (2015), the strategy that Penke (2015) describes can be connected to the Moses illusion (Reder and Kusbit, 1991), according to which neurotypical individuals are unable to detect distortions in the experimental stimuli such as “How many animals of each kind did Moses take in the Ark?.” They might fail to detect the distortion even if they do know that it was Noah and not Moses who built the Ark. This phenomenon has been explained in the literature through recognizing the existence of a *processing threshold* by means of suggesting that a partial-match strategy is operative when the stimuli is processed (Kamas et al., 1996).

Pragmatic cues are very important when the parser establishes this threshold. For example, in relation to the Moses illusion, Moses and Noah are both biblical characters and as such loosely associated in a way that can trick the parser; if Nixon was used instead of Moses, it is much more likely that the distortion would be spotted (Kamas et al., 1996). Observing that all this happens in the case of neurotypical speakers, there is no reason not to capture the problems in passivization in (a)typical speakers in the same uniform way. It has been long noted that reversible passives (e.g., “The boy is being chased by the girl” and “The girl is being chased by the boy”) are more difficult to interpret compared to non-reversible passives which are at least pragmatically odd when reversed (e.g., “The task was carried out by John” and #“John was carried out by the task”), *even in instances of typical language abilities* (Rondal, 2007). Therefore, it comes as no surprise that many atypical populations show a *selective* impairment of passives: Reversible passives are impaired, while non-reversible passives are better preserved (see Caramazza and Miceli, 1991 for aphasia). In this context, it is somewhat expected that in atypical populations that have processing limitations (and many studies attest to this for SLI; see Table 1), lower accuracy will be observed in the comprehension of some passives—not because syntax is impaired, but because the partial-match process may operate at an overall lower threshold level perturbing comprehension.

Returning to studies that put forth a syntactic impairment, Marinis and van der Lely (2007) claim that children with SLI show a particular deficit in the computational system that affects syntactic dependencies involving syntactic movement: In contrast to controls, children with SLI showed no priming effect that would indicate a filler–gap dependency. At the same time, their very high performance (ca. 90% accuracy) suggests that they were somehow able to interpret the stimuli correctly. The priming effect that the results of Marinis and van der Lely (2007) showed at the verb position indicates that an association between two different syntactic positions was indeed established, which in turns means that the ability to form such associations remains operative in SLI populations. This begs the question: Which are then the factors that lead to what many studies describe as impaired or defective syntax?

On the basis of the studies presented in Table 1, we suggest that poor memory resources (Montgomery, 2004; Bishop and Donlan, 2005), Theory of Mind deficits (Tsimpli et al., 2017b), and spell-out errors (Lin, 2007; Mastropavlou and Tsimpli, 2011) can explain why a claim for impaired syntax is put forth. For example, Schuele and Dykes (2005) argue in their longitudinal study that certain aspects of syntax may be developed late. They report omissions of infinitival *to*, wh-pronouns in clausal interrogative complements, and relative markers. Importantly, this result is cross-linguistically supported (see Mastropavlou and Tsimpli, 2011 for omissions of such functional markers in Stand ard Modern Greek). Still, one cannot conclude that such omissions occur because these syntactic nodes are broken for two reasons. The first reason is that, even if a functional element is absent, its selectional requirements are fulfilled. The findings of Mastropavlou and Tsimpli (2011) show exactly this pattern:

This leads to a paradoxical situation where the complementizer may be omitted and, hence, not merged in the syntactic position, whereas its selectional restrictions are still operative. This is particularly relevant to the omission cases of *na* which, as mentioned above, is the only complementizer which can introduce tense-dependent verb forms, i.e., the non-past, perfective form. […] We must, therefore, conclude that even in the case of omissions, children know the selectional properties imposed by C and fail to access or spell-out the required complementizer” (Mastropavlou and Tsimpli, 2011, p. 460).

The second reason boils down to the fact that such omissions are *never* consistent; the markers in question are sometimes produced and sometimes omitted within a single speaker's productions. If we accept that these omissions are due to a retrieval problem at the level of externalization, we can explain the variation observed across productions as the result of any of the following factors as well as their possible interactions:

- The presence or absence of salient pragmatic cues,
- Complexity and task-demand factors (that are related to memory limitations), and
- The (non-)salient morphophonological substance of the omitted markers (in line with the Surface Hypothesis, see Montgomery and Leonard, 1998).

If, however, the locus of impairment is the inability to construct a syntactic representation past a particular node, how is it possible that many times this syntactic representation is constructed and the problematic node surfaces intact? To give an example, if one suggests that the T(ense) node is problematic in Greek SLI, what explains that some affected persons might produce atypical realizations of T at times, while correctly producing T (and nodes past it) other times?

Having analyzed 682 linguistic profiles of children with SLI, we observe that the loci of impairment are related to externalization: morphophonology and pragmatics. Variation is attested in *some* parts of the language faculty and often appears in the form of omissions that occur due to retrieval/spell-out errors (Lin, 2007; Mastropavlou and Tsimpli, 2011), delayed mastery of phonology and failure to integrate related cues in overall processing (Kateri et al., 2005; Marshall et al., 2009), and processing, memory, and pragmatic limitations (Montgomery, 2004; Bishop and Donlan, 2005; Tsimpli et al., 2017b).

### Comparing SLI with other disorders: ASD and DS

Pragmatic difficulties and morphophonological omissions are not restricted to SLI. The literature on ASD and DS has repeatedly highlighted the existence of such features in the linguistic profiles of these populations.

#### Autism spectrum disorder (ASD)

Starting off with ASD, the Diagnostic and Statistical Manual of Mental Disorders (DSM-5; American Psychiatric Association, 2013 p. 809) defines it as “characterized by deficits in two core domains: (1) deficits in social communication and social interaction and (2) restricted repetitive patterns of behavior, interests, and activities.”

The existence of repetitive patterns is one of the key characteristics of ASD language. Kanner (1943) was the first to describe instances of “parroting,” echolalia, and atypical use of personal pronouns that involved pronoun repetition in the autistic subjects of his study. Much subsequent work has focused on pronoun reversals (i.e., use of “you” instead of “I”) in ASD and many explanations have been offered for this phenomenon, including that of echolalia (Kanner's original explanation), impaired discourse understand ing, and impaired Theory of Mind (see Brehme, 2014 for a review). Some studies have described such reversals as grammatical errors, a description that may imply a deficient language module with impaired syntax (Bartolucci and Albers, 1974; Belkadi, 2006; Wittke et al., 2017).

Apart from pronoun reversals, some ASD linguistic profiles feature other types of grammatical errors (mainly in verbal, nominal, and pronominal morphology) in around 27–28% of their utterances, a result comparable to the frequency of grammatical errors in SLI (Wittke et al., 2017). Morphology stand s out as a vulnerable domain once more, and so do pragmatic abilities in ASD (Lord and Paul, 1997; Volden et al., 2009; Marinis et al., 2013).

Syntax in ASD has received mixed descriptions. On the one hand, Bartolucci and Albers (1974, p. 131) begin their study of tense marking in autism by postulating a syntactic problem: “Certain characteristics of the syntactic structures of the language of autistic children, such as their lack of mastery of pronominalization, have been described.” On the other hand, many reviews have concluded that ASD syntax is not deficient, since many syntactic dependencies remain intact especially in high-functioning individuals, but merely follow typical development at a slower rate (Tager-Flusberg, 1981; Perovic and Janke, 2013). Other studies revealed *subtle* difficulties in some syntactic measures, regardless of language development history (Durrleman et al., 2015). Looking at the relevant results, the notion of a processing threshold that was earlier discussed in relation to SLI becomes relevant for ASD too. For instance, the ASD group in Durrleman et al. (2015) obtained lower scores in the comprehension of object relative clauses compared to subject relative clauses. This asymmetry could boil down to the non-canonical word order derived by the fronted object in object relative clauses. In other words, this additional layer of complexity could be responsible for the subject–object asymmetry that is observed in the comprehension of relative clauses not only in ASD and SLI, but also in *neurotypical* populations, with subject relatives usually being easier to process (see Carreiras et al., 2010 for a review and a counterexample).

Returning to the atypical use of pronouns, Kanner (1943) and many subsequent studies indeed offer data that involve pronoun reversals. However, they also offer examples (of the same children, at the same stage of development) that show target use of pronouns (Leivada, 2015). If these pronoun reversals were the outcome of broken syntax, how is it possible that the target performance emerges at times? Put differently, if the locus of the deficiency is to be found in the innermost component of language (i.e., syntax), what makes possible the externalization of the target pattern often in a consistent fashion?

Interestingly, use of pronouns is not always atypical in ASD. Some studies have revealed high accuracy in the comprehension of different types of pronouns including strong pronouns, clitics, and reflexives (Terzi et al., 2012, 2014 for Stand ard Modern Greek). In these studies, the lowest performance was found in the clitics condition (mean correct: 88.3%) for which the most frequent error was theta-role reversals. Is this an indication of deficient syntax? As Terzi et al. (2012, 2014) show, these children had problems with producing clitic pronouns, so it is not clear whether their low performance in the clitics condition is the result of a problem in syntactic binding or the particular grammar of clitics. Terzi et al. (2014) carried out a follow-up study that aimed to clarify this issue. The results showed that the ASD group produced a high number of clitics, yet a lower one compared to the control group (87.39% correct vs. 97.74% correct, respectively), thus favoring the scenario that renders clitics and not binding responsible for the lower performance in the clitic condition of the task.

This lower performance of the ASD group in the clitics condition is compatible with the idea put forth in the present work that loci of impairment are confined to certain parts of the language faculty. We have argued that morphology and pragmatics are shown to be vulnerable across pathologies, languages, and elicitation tasks. Clitics are markers of morphological agreement, licensed under specific pragmatic conditions, and children with ASD have troubles in ascertaining what is prominent/salient in the discourse (Terzi et al., 2016). In a subsequent study that involved narratives instead of a highly structured elicitation task, Terzi et al. (2017) found that the same group of ASD children did produce clitics, a fact that highlights the importance of the tool used to elicit data. According to Terzi et al. (2017, p. 648), ASD children “had full control of the discourse by contrast to the structured experiments, the nature of which was such that they had to take into account the discourse representation provided by the experimenter in each condition and trial.”

In this context, it could well be the case that pronoun reversals in ASD do not stem from impaired language/syntax. Studies of deaf children with autism provided below lend support for this hypothesis. It has been suggested in the relevant literature that what seems to be at stake in ASD is a *less secure anchorage in self-experience* (Lee et al., 1994). Shield and Meier's (2014) experiment is instrumental in evaluating this hypothesis. They showed deaf autistic and deaf typically developing children a picture of themselves and a picture of the experimenter. Upon seeing a picture of themselves and being asked “Who is this?,” the children with ASD either signed the pronoun “me” pointing to themselves or produced their name sign or finger-spelled their English name. In other words, they were successful both in identifying themselves and in using the correct pronoun, whenever a pronoun was used. The same strategies were employed by the typically developing group. What differentiated the two groups is not self-identification *per se* or the linguistic strategy through which self-identification was achieved, but the fact that the typically developing children “reacted with a smile or laugh and an emphatic point at his/her own body. The children with ASD had no such emotional reaction.” (Shield and Meier, 2014, p. 412). As the authors note in their discussion of these findings, forming a sense of me-ness is a key component of social behaviors such as empathy.

This less secure anchorage in me-ness can be manifested in ways that have nothing to do with the use of pronouns, thereby suggesting that the pronoun-reversal problem is not linguistic or syntactic as such (Leivada, 2015). A crucial piece of evidence that leads to this conclusion comes from studies of palm orientation during signing by deaf children with ASD. Shield and Meier (2012) found that native signers of American Sign Language with ASD showed a tendency to reverse palm orientation on signs specified for inward/outward orientation, whereas such errors were absent from the production of their typically developing peers. Observing this atypical anchorage in selfhood, one can suggest that their linguistic/grammatical counterparts (i.e., pronoun reversals) reflect not a syntactic problem but rather a more general cognitive problem that may acquire a linguistic dress (Leivada, 2015). If this observation is on the right track, syntax seems to be unimpaired in ASD, whereas other domains of language such as morphophonology (Kanner, 1943) and pragmatics (Terzi et al., 2014) stand out as particularly susceptible to impairment.

#### Down syndrome (DS)

DS is the result of a genetic abnormality most often caused from the presence of a third chromosome 21. One of the characteristics of this syndrome is atypical cognitive development. When it comes to language, our review of studies on DS suggest it is somewhat challenging when one pursues a claim of preserved syntax (as some studies have identified syntactic deficits in the profile of their subjects; e.g., Perovic, 2001).

One domain of language that has been argued to be atypical in DS is syntactic binding. Binding Theory regulates the distribution of referentially dependent elements such as anaphors and pronouns (Chomsky, 1981). Binding Principle A requires that the anaphor is locally bound by an antecedent within the same clause/domain (e.g., Mary_i_ criticized herself_i/^*^j_). Principle B requires that the antecedent of a pronoun be not in the same clause/domain as the pronoun (e.g., Bill_j_ said that John_i_ criticized him_j/^*^i_). Principle C prohibits a referential expression from being c-command ed by a coindexed element (e.g., He_i_/Bill_i_ criticized John_j/^*^i_).

Investigating the comprehension abilities of English-speaking adolescents with DS using a truth-value judgment task, Perovic (2001) found at ceiling performance on the “name-pronoun” condition (e.g., “Is Snow White washing her?”) and high performance (≥75%) for the “quantifier-pronoun” condition (e.g., “Is every bear washing him?”). This suggests that whatever the syntactic deficit amounts to, it is not Principle B. The conditions “name-reflexive” (e.g., “Is Snow White washing herself?”) and “quantifier-reflexive” (e.g., “Is every bear washing himself?”) elicited mixed responses with the percentage of correct answers ranging from 12.50 to 100% correct.

Is Principle A an example of deficient syntax in DS? The answer must be negative for a number of reasons (see Leivada, 2015 for more extensive discussion). First, would be a non-trivial task to explain why individuals with a deficient syntax would face difficulties with one binding principle but not with another, given that all binding principles require the same underlying grammatical knowledge (Perovic, 2001). Second, the results did not show a unanimous pattern of Principle A violations. The average number of correct responses on the “name-reflexive” condition was above chance (56.56% correct). In turn, the average number of correct responses on the “quantifier-reflexive” condition was below chance (35.94% correct), but as Perovic (2001) noted, two participants showed very poor performance even on the control condition that involved quantified NPs and no anaphors. It is then possible that these participants had issues with quantification generally, which resulted in errors on some of the tested conditions.

Tsakiridou (2006) and Christodoulou (2011) focused on Standard Modern Greek and Cypriot Greek DS grammars, respectively. Both showed that the deviations noted in the DS linguistic profile were related to morphophonology: non-target morphological markings (Tsakiridou, 2006) and phonetically or morphophonologically conditioned differences (Christodoulou, 2011). Pragmatics in DS is also atypical. Challenges may include initiation of topics and communicative repairs and aspects of narratives (Martin et al., 2009).

The overall picture that emerges with respect to the linguistic phenotype of DS is one that supports the claim that the aspects of language which appear to be atypical are related to specific parts of the language faculty: morphophonology and pragmatics.

### The locus preservation hypothesis

Having reviewed the literature on three developmental disorders, the first observation is that certain morphological markers reveal themselves as highly susceptible to impairment (e.g., agreement markers and clitics). Second, syntax appears to be preserved. Undoubtedly, some studies have identified problems in the comprehension or production of complex syntactic structures across disorders (see Table 1 for SLI). Yet, when considering the general processing limitations that are arguably present in the pathologies discussed in the present work (even though, unfortunately, not fully or equally measured in all studies), we are facing a classical chicken and egg question (Bishop and Donlan, 2005): Do syntactic limitations lead to conceptual and memory limitations or do conceptual and memory limitations result in an atypical syntax?

We have argued that poor memory resources (Montgomery, 2004; Bishop and Donlan, 2005), Theory of Mind deficits (Tsimpli et al., 2017b), and retrieval/spell-out errors (Lin, 2007; Mastropavlou and Tsimpli, 2011) can explain why a claim for impaired syntax is put forth at times. Observing how “linguistic” deficits such as the incorrect use of anaphors in ASD can derive from a general cognitive problem in establishing me-ness in relation to the outer world, we tentatively conclude that atypical cognitive abilities (i.e., processing impairments, memory limitations; see Table 1) may result in what looks as an atypical syntax. The latter is manifested mainly through omissions, and recall that it would be wrong to conclude that such omissions occur because the related syntactic nodes or operations are broken. The selectional requirements of omitted functional elements may still be operative and satisfied (Mastropavlou and Tsimpli, 2011). Therefore, it makes more sense to describe such omissions as spell-out errors related to the externalization component of language.

Looking at the distribution of impaired and preserved markers/levels of linguistic analysis, variation across pathologies can by formally captured within the *Locus Preservation Hypothesis* (see also Leivada, 2015 for an earlier formulation based on Greek data only):

(1) *Locus Preservation Hypothesis*

Syntactic operations are preserved and impenetrable to variation across developmental pathologies.

**Figure 2:**
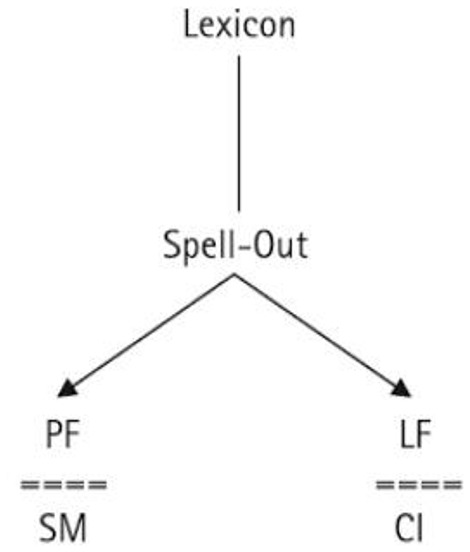
A minimalist architecture of the grammar (Tsimpli et al., 2017a, p. 494).

Assuming a widely accepted architecture of the grammar as the one shown in Figure 2, the Locus Preservation Hypothesis holds that the computational part of the human language faculty is invariably preserved, with the operations (internal and external) Merge and Agree applying in an intact manner all the way to constructing the internal interface levels of Logical Form (LF) and Phonetic/Phonological Form (PF).

The purported pragmatic deficiencies (Katsos et al., 2011) arise post-syntactically, where the conceptual-intentional system (CI) is accessed along with pragmatic information and encyclopedic/world knowledge. Likewise, the externalization difficulty observed in language production tasks and spontaneous speech (Mastropavlou and Tsimpli, 2011) is relevant at the other interface, the articulatory-perceptual or sensory-motor system (SM). The fact that bound morphophonological building blocks are often misused (Bedore and Leonard, 2001) suggests the need for a finer distinction of the “Lexicon” than what the architecture in Figure 2 allows.

**Figure 3:**
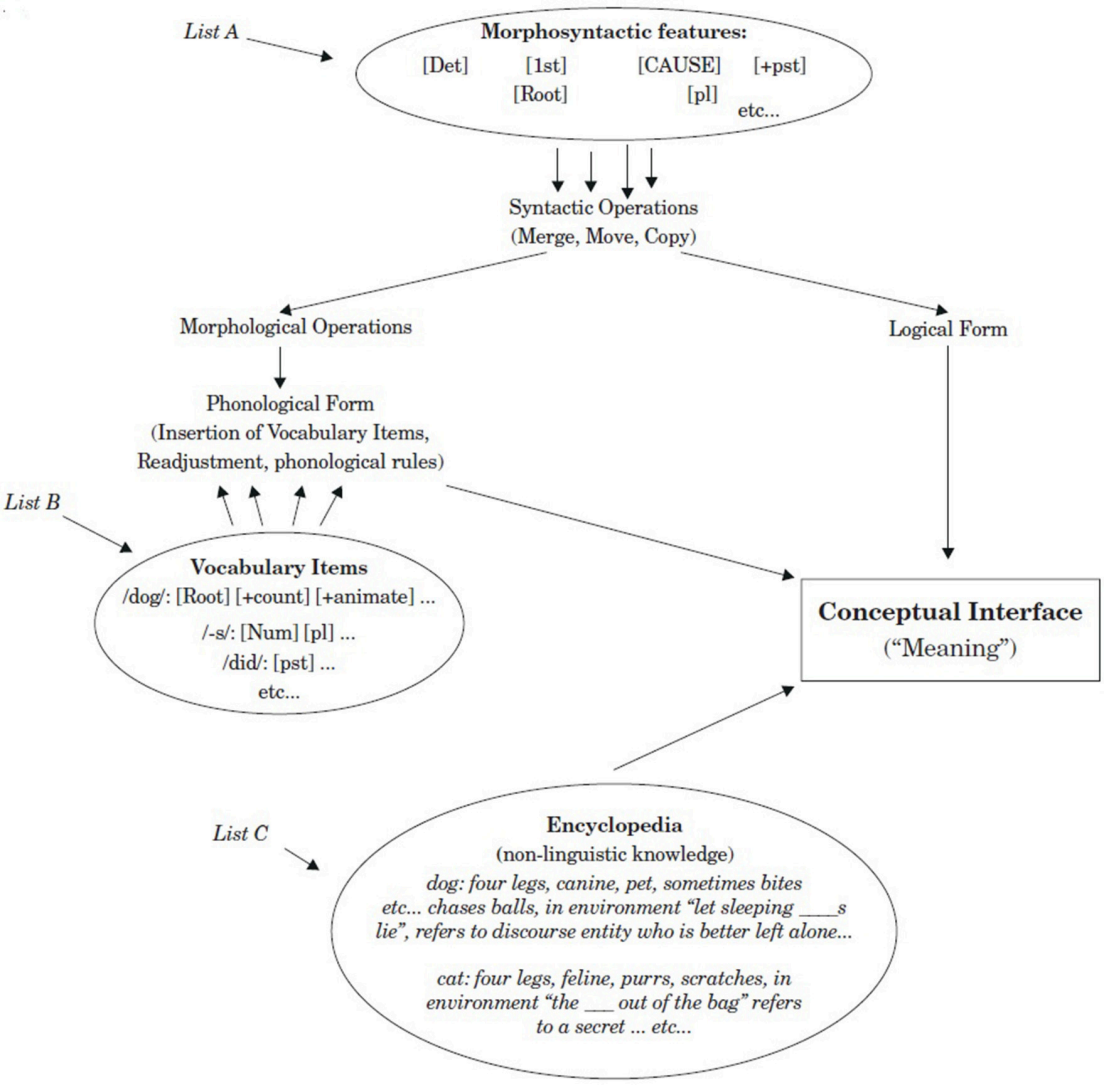
Distribution of labor in distributed morphology (Harley and Noyer, 1999, p. 3).

Mapping the Locus Preservation Hypothesis to the distribution of labor put forth in Distributed Morphology, it seems that the first set of operations in the transition from List A to List B are resilient to impairment across atypical cognitive phenotypes. In contrast, morphophonological operations and encyclopedic knowledge are consistently susceptible to impairment across atypical cognitive phenotypes (Leivada, 2015). The results that led to this conclusion come from three developmental disorders (SLI, ASD, DS), but there are reasons to believe that this conclusion would hold even when one examines the linguistic profile of acquired pathologies such as aphasia; a topic to be pursed in future work on the Locus Preservation Hypothesis. A more detailed model is provided in Figure 3.

Overall, based on our review of different research studies, not all pathologies show the same impaired markers—but the same markers are consistently impaired across pathologies. The Locus Preservation Hypothesis is thus pathology-independent and can be used to support cross-linguistic findings.

The important question is why syntactic operations are better preserved in a consistent way across disorders with different genetic etiology. One explanation is that the phenotypic overlaps that we identified are in fact surface reflections of more deeply rooted overlaps at the connectome or even the oscillome (Benítez-Burraco and Murphy, 2016). Observing that the hierarchy of brain oscillations has remained remarkably preserved during mammalian evolution (Buzsáki et al., 2013), Benítez-Burraco and Murphy (2016) suggest that language deficits in various cognitive disorders can be traced back to a brain syntax network. In this context, it can be argued that syntax is preserved because it is implemented through a network that is less novel in evolutionary terms, hence more resilient to impairment. Less resilient networks underlie cognitive capacities more recently evolved in phylogenetic terms, whereby selective pressures have not yet given rise to the development of robust compensatory mechanisms (Toro et al., 2010; Murphy and Benítez-Burraco, 2016). This claim grants support to another hypothesis recently explored in the language evolution literature: the “syntax-before-phonology” hypothesis. Based on a review of linguistic calls across species, Collier et al. (2014) argue that syntax, which is universally present in all languages, possibly evolved before phonology, since many systems of communication in other species have the former but not the latter. The Locus Preservation Hypothesis suggests that phonology is less resilient in stark contrast to syntax—a finding that is in line with what the ethological record reveals (Collier et al., 2014).

## Conclusions

The present work has put forth a novel hypothesis: the Locus Preservation Hypothesis, in order to capture the distribution of what are considered atypical linguistic markers across different languages and pathologies. It has been argued that syntactic operations are resilient to impairment across developmental disorders; in contrast, morphophonology and pragmatics are consistently impaired. This conclusion stand s in agreement with a long line of literature that discusses overlaps in the behavioral profile of populations with different pathologies, both acquired and developmental (Phillips, 2005; Glisky, 2007; Kambanaros and van Steenbrugge, 2013; Benítez-Burraco and Boeckx, 2014; Leivada, 2014, 2015; Kambanaros and Grohmann, 2015; Tsimpli et al., 2017a).

The Locus Preservation Hypothesis can gain more support by expanding the range of languages and pathologies that are examined. Once this is done, the following question to be explored in detail is why syntax would be preserved. One explanation we contemplated in the present work relates to the possibility of an underlying uniform etiology across the reviewed disorders. This uniformity can be traced back to brain network organization (van den Heuvel and Sporns, 2013; Crossley et al., 2014; Benítez-Burraco and Murphy, 2016). Addressing the parallels that can be observed across different levels of representation (phenome, connectome, dynome, and oscillome) from a phylogenetic perspective, we have established a connection between the hypothesis put forth in the present work and the “syntax-before-phonology” hypothesis of Collier et al. (2014): Syntax is better preserved because it evolved before other domains of language (e.g., morphology and phonology). Therefore, syntax had more adaptation time for the development of compensatory mechanisms, unlike more recently evolved cognitive/linguistic capacities. Future research on the Locus Preservation Hypothesis will elaborate on the syntax-first hypothesis and flesh out the connections between the observed overlap at the phenotypic level and its roots in deeper levels of representation.

## Author contributions

All authors participated in the analysis. EL drafted the manuscript. MK and KG reviewed and revised the manuscript.

### Conflict of interest statement

The authors declare that the research was conducted in the absence of any commercial or financial relationships that could be construed as a potential conflict of interest.
